# Supplementary material for: Age-related erosion of X chromosome inactivation in human tissues
Source: bioRxiv. 2026 Jul 1:2025.12.09.693282. Preprint. [Version 2] doi: 10.64898/2025.12.09.693282 (PMC13345331; doi:10.64898/2025.12.09.693282)
Supplement: Supplement 3 [file NIHPP2025.12.09.693282v2-supplement-3.pdf]

bioRxiv preprint doi: https://doi.org/10.64898/2025.12.09.693282; this version posted July 1, 2026. The copyright holder for this preprint (which was not certified by peer review) is the author/funder. This article is a US Government work. It is not subject to copyright under 17 USC 105 and is also made available for use under a CC0 license.

Supplementary Figure Legends

Supplementary Figure 1 | Variance partitioning of allelic expression

Top. Boxplots of the proportion of variance in allelic expression explained per gene (y-axis) by each predictor variable (x-axis). Each gene has one point in each boxplot, and these values add up to 1. The mean proportion of variance explained by each predictor across all genes is shown below each boxplot. All samples are included.

Bottom. Same as the top plot, but for the N=81 nmXCI samples only.

Supplementary Table Legends

Supplementary Table 1 | Linear modelling results

For each gene, the linear modelling results across 32 models are shown. Predictors included in each model are indicated in Columns D-H. Numbers of samples, participants, and tissues are provided in Columns J-L. Model run information is in Column N (lm = linear model; lmer = linear mixed model; lmer_singular = linear mixed model with singular fit; skipped_n_ind<=3 = model was skipped because of too few individuals; failed = model failed to run). Model results (for the age effect) are in Columns P-W. Model comparisons are in Columns Y-AB).

Supplementary Table 2 | Comparison across analysis of the bulk nmXCI dataset

For each gene, the results from the prior consensus, matched tissue, and linear modelling analyses are summarized. Linear modelling results reflect those from best fit models (Methods). “Mixed” in prior consensus = escaping in aged and young samples. “Mixed” in matched tissue = mixed pattern across tissues. “No switch” = no age difference in XCI status across tissues. NA = gene was not included in the analysis.

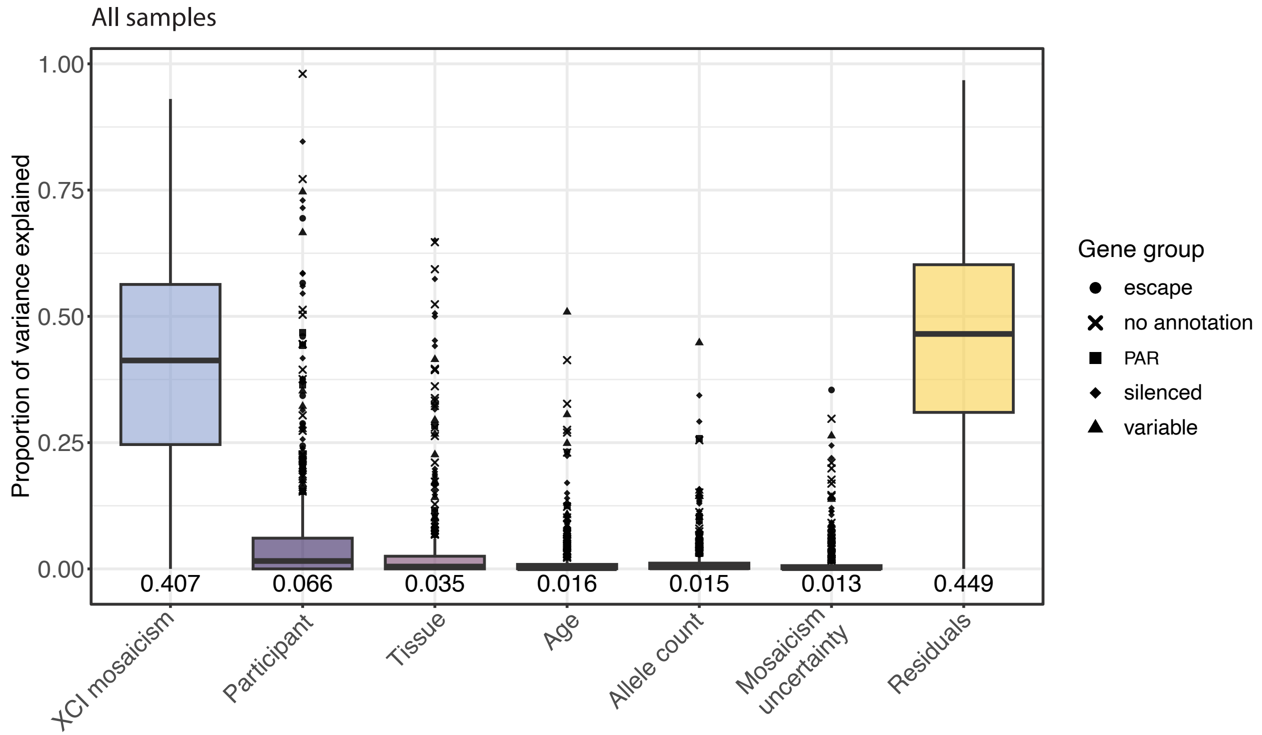
All samples
Proportion of variance explained
1.00
0.75
0.50
0.25
0.00
0.407
0.066
0.035
0.016
0.015
0.013
0.449
XCI mosaicism
Participant
Tissue
Age
Allele count
Mosaicism uncertainty
Residuals
Gene group
escape
no annotation
PAR
silenced
variable

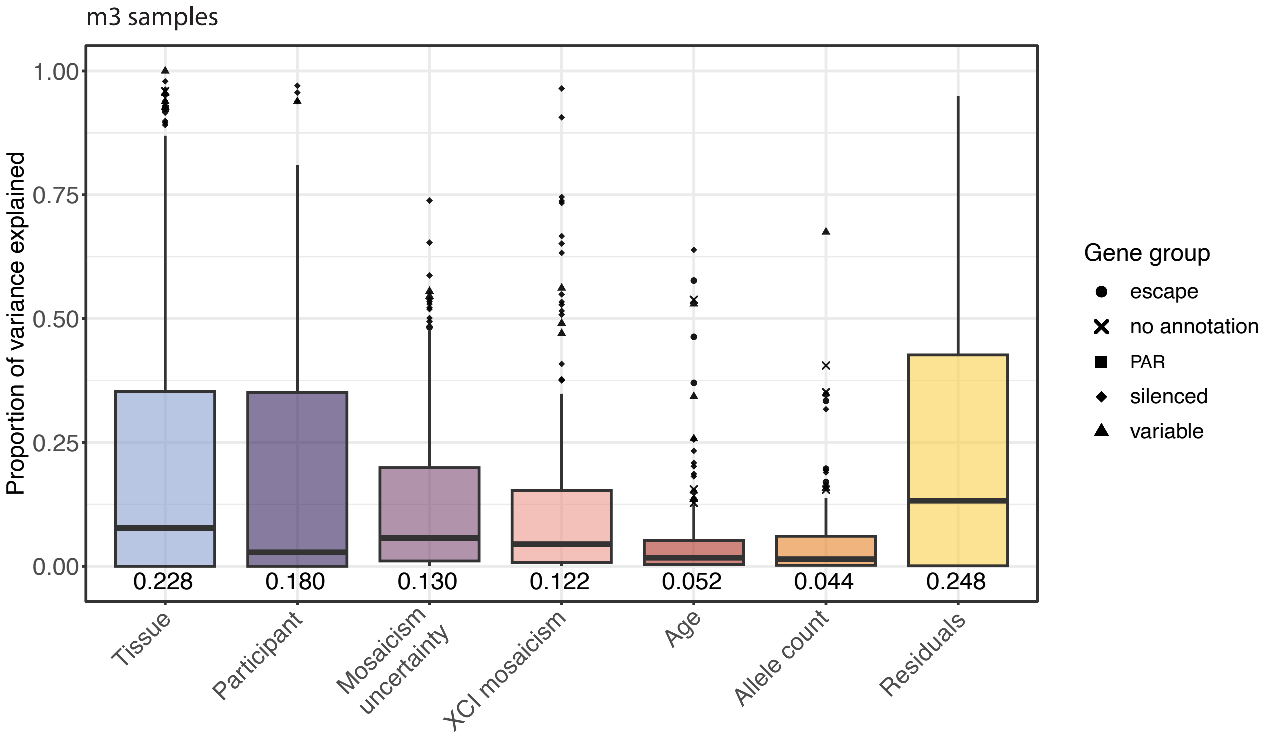
m3 samples
Proportion of variance explained
1.00
0.75
0.50
0.25
0.00
0.228
0.180
0.130
0.122
0.052
0.044
0.248
Tissue
Participant
Mosaicism uncertainty
XCI mosaicism
Age
Allele count
Residuals
Gene group
escape
no annotation
PAR
silenced
variable
